# Assessment of the Gut Microbiome in Patients with Coexisting Irritable Bowel Syndrome and Chronic Fatigue Syndrome

**DOI:** 10.3390/nu17132232

**Published:** 2025-07-05

**Authors:** Marcin Chojnacki, Aleksandra Błońska, Aleksandra Kaczka, Jan Chojnacki, Ewa Walecka-Kapica, Natalia Romanowska, Karolina Przybylowska-Sygut, Tomasz Popławski

**Affiliations:** 1 Department of Clinical Nutrition and Gastroenterological Diagnostics, Medical University of Lodz, 90-647 Lodz, Poland; mchojnacki@csk.umed.pl (M.C.); aleksandra.blonska@umed.lodz.pl (A.B.); aleksandra.kaczka@umed.lodz.pl (A.K.); jan.chojnacki@umed.lodz.pl (J.C.); 2 Department of Gastroenterology, Medical University of Lodz, 92-213 Lodz, Poland; ewa.walecka-kapica@umed.lodz.pl (E.W.-K.); natalia.romanowska1@stud.med.lodz.pl (N.R.); 3 Department of Pharmaceutical Microbiology and Biochemistry, Medical University of Lodz, 92-215 Lodz, Poland; karolina.przybylowska@umed.lodz.pl

**Keywords:** irritable bowel syndrome, chronic fatigue syndrome, gut microbiome, tryptophan metabolism, kynurenine pathway, LC-MS/MS

## Abstract

**Background:** The gut microbiome is a key modulator of the gut–brain axis and may contribute to the pathophysiology of both gastrointestinal and systemic disorders. This study aimed to evaluate gut microbiota composition and tryptophan/phenylalanine metabolism in women with unclassified irritable bowel syndrome (IBS-U), with or without coexisting chronic fatigue syndrome (CFS). **Methods:** Eighty women were enrolled and divided into two groups: IBS-U without CFS (Group I, n = 40) and IBS-U with coexisting CFS (Group II, n = 40). Microbial composition and diversity were assessed using the GA-map™ Dysbiosis Test, including the dysbiosis index (DI) and Shannon Diversity Index (SDI). Hydrogen and methane levels were measured in breath samples. Urinary concentrations of selected microbial and neuroactive metabolites—homovanillic acid (HVA), 5-hydroxyindoleacetic acid (5-HIAA), kynurenine (KYN), kynurenic acid (KYNA), xanthurenic acid (XA), quinolinic acid (QA), hydroxyphenylacetic acid (HPA), and 3-indoxyl sulfate (3-IS)—were quantified using LC-MS/MS. Fatigue severity was assessed using the Chalder Fatigue Questionnaire (CFQ-11) and the fatigue severity scale (FSS). **Results:** Compared to Group I, patients with IBS-CFS showed significantly greater microbial diversity, higher breath methane levels, and elevated urinary concentrations of QA, XA, 3-IS, and HVA, alongside lower concentrations of 5-HIAA and KYN. Fatigue severity was positively correlated with urinary XA and QA levels. **Conclusions:** Women with IBS and coexisting CFS exhibit distinct gut microbiota and tryptophan metabolite profiles compared to those without fatigue. The observed metabolite–symptom associations, particularly involving neuroactive kynurenine derivatives, warrant further investigation. These preliminary findings should be interpreted as hypothesis-generating and require validation through high-resolution microbiome analyses, functional pathway profiling, and longitudinal or interventional studies to clarify causality and clinical significance.

## 1. Introduction

### 1.1. Chronic Fatigue Syndrome: Historical Perspective and Diagnostic Evolution

Chronic fatigue in various diseases has been described in medical literature since the early 20th century. In 1956, the term Myalgic Encephalopathy (ME) was proposed, primarily as an inflammatory neurological disease characterized by prolonged muscle fatigue and sensory and cognitive symptoms [1,2]. In 1988, Holmes [3] was the first to use the term chronic fatigue syndrome (CFS) as a consequence of mononucleosis. In 1994, Fukuda et al. [4] proposed diagnostic criteria for this syndrome, including a sore throat, tender lymph nodes, muscle pain, multi-joint pain, headaches, unrefreshing sleep, and post-exertional malaise. Additionally, persistent (at least 6 months) or intermittent chronic fatigue with relapses is not a symptom of another disease. In 2003, these differentiated definitions were introduced for both conditions, with the Canadian Consensus Criteria defining the mixed condition (ME/CFS). As a result, ME and CFS were recognized as similar diseases with overlapping symptoms [5]. However, many researchers and clinicians considered ME and CFS to be distinct disorders and felt that they could not be replaced by a hybrid diagnosis. ME was primarily associated with inflammatory changes in the nervous system of various etiologies, while CFS was classified as a psychosomatic disease. In 2015, the US National Academy of Medicine recommended a new definition, replacing the hybrid ME/CFS with Systemic Exertion Intolerance Disease [6]. The idea of inflammatory etiology for these syndromes has resurfaced during the COVID-19 pandemic, given that the chronic effects of this infectious disease exhibit symptoms similar to ME/CFS [7]. However, their pathogenesis remains unclear and requires further research.

### 1.2. Gut Microbiome Alterations in Chronic Fatigue Syndrome

Particular attention has been directed toward changes in the gut microbiome, which are thought to be involved in the pathogenesis of many systemic diseases. Dysbiosis has already been detected in several CFS studies [8,9], although a specific, consistent microbiome composition has yet to be identified [10,11]. A reduction in bacterial diversity and richness, along with lower levels of anti-inflammatory species and higher levels of pro-inflammatory bacterial species, such as *Enterobacteriaceae*, has been observed in stool samples [11]. Other researchers have also noted decreased levels of beneficial *Bifidobacteria*, diminished anti-inflammatory species of Firmicutes, and increased abundance of *Enterobacteriaceae* and *Clostridium* spp. [9,12]. Another study has shown decreased levels of anti-inflammatory Firmicutes [13,14]. The results from these studies were obtained using different methods and without thorough analysis of dietary intake, making comparisons difficult. However, prevailing opinion suggests that the high abundance of pro-inflammatory bacteria may lead to alterations in the intestinal barrier and their translocation into the bloodstream [15,16,17,18]. These changes provoke an immune response and result in systemic inflammation. In patients with gut dysbiosis, elevated levels of antibodies against bacterial and food antigens are observed [18,19]. Dysbiosis is also indicated by alterations in various bacterial products and metabolites. Reduced abundance of SCFA-producing bacteria, such as *Faecalibacterium*, or certain *Firmicutes* spp. and *Bacteroidetes* spp., has been demonstrated in ME/CFS patients [19]. SCFAs are considered regulators in the neuroimmunological system and are associated with anti-inflammatory species [8,10], although they can also have neurotoxic effects [20].

### 1.3. Tryptophan Metabolism and the Microbiota–Gut–Brain Axis

Symptoms similar to CFS occur in other chronic health disorders, including serotonin deficiency syndrome and dopamine deficiency syndrome. For this reason, the cause of chronic fatigue was sought in the homeostasis of these neurotransmitters, but no clear confirmation of the CFS hypothesis was obtained [21]. The substrate for the synthesis of serotonin is L-tryptophan (TRP), which is metabolized in more than 90% in the gastrointestinal tract along the serotonin, kynurenine, and indole pathways. Serotonin does not cross the blood-brain barrier, so its serum level is not an indicator of TRP levels in the brain. Currently, more attention is given to the study of TRP metabolites in urine, as they reflect TRP metabolism in both peripheral tissues and the CNS [22]. Most TRP is metabolized along the kynurenine pathway, involving indoleamine 2,3-dioxygenase (IDO-1). This pathway produces several metabolites, such as kynurenine, kynurenic acid, xanthurenic acid, quinolinic acid, and others, which are thought to have neurotoxic effects [23]. The results concerning these metabolites in patients with CFS are inconsistent. IDO is induced by pro-inflammatory cytokines, which increase the degradation of TRP to kynurenines, altering immunological tolerance towards chronic infections. Some researchers speculate (metabolic trap hypothesis) that insufficient kynurenine production results in abnormal serotonin synthesis. On the other hand, it has been shown that inflammatory factors are a common trigger of CFS by inducing IDO-1 to produce more kynurenine [24]. Other researchers believe that abnormal immune activation and intensified kynurenine metabolism might play a significant role only in the initiation of CFS [25,26]. It should be noted that TRP is also largely metabolized by intestinal bacteria via the indole pathway. The enzymes that initiate these metabolic pathways compete for access to TRP, and depending on their activity, these results may change over time [23]. Tryptophan and phenylalanine (PHA) metabolism are influenced by many factors, including dysbiosis [27,28]. Intestinal bacteria may contribute to the production of these metabolites through mechanisms that remain to be elucidated, and such contributions cannot be confirmed without species-level resolution or functional pathway analysis. This may reflect in dysfunction of the gastrointestinal tract and other organs, including the central nervous system [29,30,31]. Due to the multitude of processes taking place, the intestines have an extensive network of neurons connected to the brain via the gut–brain axis, with the participation of neurotransmitters. Bacteria may play a role in these regulations, and for this reason, the term microbiota–gut–brain axis is appropriately used [32,33]. However, the results of many studies are not consistent, especially in cases of coexisting functional diseases of the gastrointestinal tract and chronic fatigue syndrome [19,33], which require further research.

### 1.4. Study Rationale and Objectives

This study aimed to assess the gut microbiota profile and the metabolism of tryptophan and phenylalanine in women with unclassified IBS, both with and without coexisting CFS. We hypothesized that patients with coexisting CFS would exhibit distinct microbiome characteristics and elevated levels of neurotoxic tryptophan metabolites, which we expected would correlate with the severity of fatigue.

## 2. Materials and Methods

### 2.1. Participants and Study Design

This study enrolled 80 women aged 29 to 60 years (mean age: 47.1 ± 11.2), recruited between 2019 and 2024 at the Department of Gastroenterology and the outpatient clinic of the Central Hospital of the Medical University of Lodz representing a predominantly perimenopausal population. All participants reported abdominal complaints, while some also reported fatigue, mood disturbances, or sleep difficulties. The initial diagnostic workup aimed to exclude organic gastrointestinal, neurological, and psychiatric conditions. This included comprehensive laboratory tests, endoscopic examinations, and histological assessment of duodenal and colonic mucosa. Based on symptom presentation and the Rome IV criteria [34,35], all participants were diagnosed with unclassified irritable bowel syndrome (IBS-U). This subtype applies to patients who report IBS-related symptoms but do not meet criteria for the other subtypes (IBS-C, IBS-D, and IBS-M), typically due to a variable or inconsistent stool pattern. After IBS-U diagnosis, participants underwent a standardized assessment of mental health and chronic fatigue severity. Based on these evaluations, they were assigned to one of two study groups:

- Group I (n = 40): patients with IBS-U and no chronic fatigue,
- Group II (n = 40): patients with IBS-U and coexisting chronic fatigue syndrome (IBS-CFS), meeting established diagnostic criteria.

Both groups were invited to participate in additional analyses evaluating gut microbiota composition and metabolism of tryptophan and phenylalanine. Exclusion criteria included: age over 60 years; diagnosis of inflammatory bowel disease, hepatic, pancreatic, renal, or thyroid disorders; lactose or gluten intolerance; malnutrition; obesity; or use of hormonal contraceptive or hormone replacement therapy within 3 months prior to study entry, probiotics, or psychotropic medications within the previous three months. No participants were taking medications known to significantly influence tryptophan metabolism or microbiome composition.

### 2.2. Fatigue Assessment

Chronic fatigue was assessed using two validated instruments: the Chalder Fatigue Questionnaire (CFQ-11) and the fatigue severity scale (FSS).

The CFQ-11 is a widely used screening tool in clinical and epidemiological studies to evaluate the presence and intensity of fatigue-related symptoms [36]. It assesses both the physical and mental components of fatigue, including items related to cognitive impairment, myalgia, arthralgia, headache, sleep disturbances, sore throat, lymphadenopathy, and post-exertional malaise lasting more than 24 h. Each item is rated on a 4-point Likert scale, resulting in a total score ranging from 11 to 44. Higher scores indicate greater fatigue severity. The CFQ-11 was used to support the diagnosis of chronic fatigue syndrome (CFS) following established symptom thresholds. To further quantify the impact of fatigue on daily functioning, participants also completed the fatigue severity scale (FSS) [37]. This instrument is commonly used in the assessment of neurological and rheumatological disorders, such as multiple sclerosis and systemic lupus erythematosus, where fatigue is a prominent clinical feature. It consists of nine items addressing motivation, physical endurance, and the extent to which fatigue interferes with social, occupational, and personal activities. Each item is scored on a 7-point scale (1 = strongly disagree, 7 = strongly agree), resulting in a total score ranging from 9 to 63. Higher scores reflect more disabling fatigue. Both tools were administered before group allocation and served to identify participants who met criteria for inclusion in the IBS-CFS group.

### 2.3. Laboratory Tests

All participants underwent standard laboratory evaluations to exclude potential confounding metabolic, endocrine, or inflammatory conditions. The tests included measurements of hemoglobin, glycated hemoglobin (HbA1c), lipid profile, bilirubin, serum iron, vitamin D_3_, vitamin B_12_, folic acid, magnesium, potassium, urea, creatinine, and thyroid function markers, including thyroid-stimulating hormone (TSH), free thyroxine (fT_4_), and free triiodothyronine (fT_3_). Additionally, serological screening for celiac disease was conducted using antibodies to tissue transglutaminase (anti-tTG) and deamidated gliadin peptide (anti-DGP). Liver, kidney, and pancreatic function markers were assessed, along with systemic inflammatory status (as indicated by C-reactive protein, CRP) and intestinal inflammation (as measured by fecal calprotectin). Urine samples were collected in the morning, after overnight fasting, into dedicated containers pre-filled with 0.1% hydrochloric acid solution to stabilize indole compounds. Analysis focused on a panel of microbial-derived organic acids, including p-hydroxyphenylacetic acid (HPA), 3-indoxyl sulfate (3-IS, also known as indican), and hippuric acid (HA). A total of twelve organic acids were quantified using the Organix Neuro and Organix Gastro diagnostic panels (ALAB Laboratories, Warsaw, Poland). Measurements were performed using liquid chromatography coupled with tandem mass spectrometry (LC–MS/MS) on a Shimadzu Nexera system with fluorescence detection (Kyoto, Japan; software: Solution version 4.50). Metabolite concentrations were normalized to urinary creatinine and expressed as milligrams per gram of creatinine (mg/gCr). The concentrations of these metabolites reflect microbial fermentation and amino acid degradation processes in the gut and were therefore used as indirect markers of intestinal microbiota composition and metabolic activity.

### 2.4. Breathing Test

To minimize potential confounding factors, all participants were instructed to avoid antibiotics and probiotics for at least four weeks prior to the test. In addition, for seven days preceding the procedure, a low-fermentation diet was implemented, and any fiber supplementation was discontinued.

The hydrogen–methane breath test (HMBT) was conducted using the GastroCH_4_ECK Gastrolyzer device (Bedfont Ltd., Harrietsham, UK), with data analyzed using GastroCHART™ software (www.bedfont.com 2021). The procedure adhered strictly to the North American Consensus Guidelines for Breath Testing [38], which provide standardized protocols for lactulose-based tests used in the evaluation of small intestinal bacterial overgrowth (SIBO).

Participants fasted overnight (minimum 12 h) before baseline sampling. After baseline breath collection, each subject ingested a 10 g dose of lactulose dissolved in 200 mL of water, consistent with consensus recommendations for substrate loading. Breath samples were subsequently collected every 15 min over a 3 h period. Measurements of hydrogen (H_2_) and methane (CH_4_) levels were taken, with primary statistical analyses focusing on values at 0, 90, and 180 min.

According to consensus criteria, a rise in hydrogen concentration ≥ 20 parts per million (ppm) above baseline within the first 90 min is considered indicative of SIBO. Similarly, a methane concentration ≥ 10 ppm at any time point suggests the presence of methane-producing archaea. Elevation of both gases was interpreted as mixed-type overgrowth. While the standard evaluation interval for SIBO is 90 min, the extended 180 min sampling window in our protocol allowed assessment of colonic fermentation and differentiation between proximal small intestinal overgrowth and distal gut microbial activity.

Gas detection was performed using calibrated instrumentation with a detection threshold of 1 ppm for both gases, ensuring high sensitivity and reproducibility. These breath measurements served as indirect functional indicators of gastrointestinal microbial fermentation and were correlated with microbiota composition metrics to provide a comprehensive overview of gut microbial activity in vivo.

The hydrogen–methane breath test was performed on the same day as fecal sample collection, following standardized pre-test dietary restrictions. All participants adhered to a low-fermentation diet and discontinued fiber supplements for at least seven days prior to testing, as recommended to minimize background microbial fermentation and intestinal gas variability.

### 2.5. Dysbiosis Test

Gut microbiota composition and dysbiosis were assessed using the GA-map™ Dysbiosis Test (Oslo, Norway), a clinically validated, commercially available assay developed by Genetic Analysis AS. The test methodology follows the protocol described initially by Casén et al. [39], and was performed externally by ALAB Laboratories (Warsaw, Poland), which uses the complete proprietary system purchased from the manufacturer.

The test employs 54 DNA probes targeting bacterial 16S rRNA gene sequences within seven variable regions (V3–V9). These probes are specific to different bacterial taxa, allowing for the simultaneous detection of over 300 bacterial groups at various taxonomic levels. The assay is run on the Luminex MAGPIX platform, and data are processed using the GA-map™ Analyzer Software v1.4, which generates a dysbiosis index (DI) score based on a proprietary algorithm fully embedded in the licensed software.

The dysbiosis index (DI) is a composite score ranging from 1 to 5 that indicates the degree of deviation from a normobiotic (healthy) reference profile. A DI ≤ 2 indicates a normal (non-dysbiotic) microbiota composition, while a DI > 2 indicates the presence of dysbiosis. Importantly, this algorithm and all DI calculations were performed automatically by the GA-map system, as implemented by ALAB; the study team performed no additional interpretation or modification. Additionally, Shannon alpha diversity was calculated using the same platform to provide a measure of microbial richness and evenness. The GA-map Dysbiosis Test in our study included phyla consistent with current taxonomic nomenclature, covering Actinomycetota (formerly Actinobacteria), Bacteroidota (formerly Bacteroidetes), Bacillota (formerly Firmicutes), Pseudomonadota (formerly Proteobacteria), Mycoplasmatota (formerly Tenericutes), and Verrucomicrobiota. These analyses were performed using the FloraGen panel, a branded implementation of the GA-map test offered by ALAB.

While more detailed microbiome sequencing methods (e.g., 16S rRNA gene sequencing or shotgun metagenomics) exist, the GA-map™ Dysbiosis Test was selected for this study due to its clinical validation, standardized methodology, and established dysbiosis scoring system. These features made it a pragmatic and appropriate choice for an exploratory investigation aimed at characterizing broad microbial patterns in a clinical cohort. Nonetheless, we acknowledge its limitations in resolving microbial composition at the species level or providing insight into functional gene expression.

Fecal samples were collected by participants on the morning of the scheduled breath test, using sterile, sealed containers provided in advance. Samples were delivered in person and stored at ambient temperature (20–22 °C) for up to 7 days before analysis, according to the GA-map™ Dysbiosis Test manufacturer’s instructions. This protocol aligns with validated clinical procedures for this assay.

### 2.6. Nutritional Recommendations

To minimize the influence of lifestyle and dietary factors on microbiome composition and metabolite levels, all participants followed a standardized nutritional protocol. A habitual, nutritionally balanced diet was maintained throughout the study, with a target intake of approximately 2000 kcal/day and macronutrient distribution standardized across both groups (minimum daily intake: protein—50 g; carbohydrates—270 g; fat—70 g; soluble fiber—30 g).

In preparation for breath and urine sample collection, dietary intake of key amino acids was restricted for three days prior to testing: phenylalanine intake was limited to ≤40 mg/kg/day, and tryptophan to ≤15 mg/kg/day. This pre-analytical control was designed to reduce interindividual variability in amino acid-derived microbial and neuroactive metabolites.

Compliance with dietary recommendations was monitored using supervised dietary logs, reviewed by trained dietitians in regular contact with participants (via telephone and email). Nutrient intake, including amino acid content, was analyzed using the Kcalmar. Pro-Premium dietary software (https://kcalmar.com 2024, Hermex, Lublin, Poland).

Additionally, to reduce confounding factors, individuals who had used probiotics, antibiotics, or other medications known to affect the microbiome or amino acid metabolism within three months prior to enrollment were excluded from the study.

These methodological measures were implemented to control for potential lifestyle and dietary confounders and to enhance the interpretability of observed microbiome and metabolite profiles.

### 2.7. Ethical Issues

This study was conducted as an open-label clinical trial in compliance with the principles of the Declaration of Helsinki and the International Council for Harmonization Guidelines for Good Clinical Practice (ICH-GCP). Ethical approval was obtained from the Bioethics Committee of the Medical University of Lodz (approval number: RNN/176/18/KE). Written informed consent was obtained from all participants before enrollment.

### 2.8. Data Analysis

All statistical analyses were conducted using STATISTICA version 13.3 (TIBCO Software Inc., Palo Alto, CA, USA). The distribution of continuous variables was assessed using the Shapiro–Wilk test to determine the appropriate statistical tests for group comparisons. For variables with a normal distribution, differences between the IBS-U (Group I) and IBS-CFS (Group II) groups were analyzed using the independent samples Student’s *t*-test. For non-normally distributed variables, the Mann–Whitney U test was applied. Data are presented as mean ± standard deviation (SD) for normally distributed variables and as median with interquartile range (IQR) for skewed distributions, unless otherwise specified. Categorical variables, such as the presence or absence of specific clinical symptoms or relative bacterial abundance, were compared between groups using the two-proportion Z-test. Correlations between continuous variables—including fatigue severity scores, urinary concentrations of phenylalanine- and tryptophan-derived metabolites, hydrogen and methane concentrations at selected breath test time points (0, 90, and 180 min), and gut microbiota indices such as the dysbiosis index (DI) and Shannon Diversity Index (SDI)—were evaluated using Spearman’s rank-order correlation coefficient (rho, ρ). All statistical tests were two-tailed, and a *p*-value of less than 0.05 was considered indicative of statistical significance.

## 3. Results

### 3.1. Participant Demographic Assessment

The participants were employed across various professional sectors, including public trading (32.5%), education (28.75%), medical services (22.5%), banking (10.0%), and other service industries (21.25%). The mean age was 47.9 ± 11.4 years in Group I (IBS-U) and 44.6 ± 12.3 years in Group II (IBS-CFS), with no statistically significant difference between the groups (*p* > 0.05). Regarding marital status, 64 participants were married, while the remaining individuals were single. The nutritional status, assessed by body mass index (BMI), was similar in both groups: 23.1 ± 1.1 kg/m^2^ in Group I and 22.9 ± 1.2 kg/m^2^ in Group II (*p* > 0.05).

### 3.2. Assessment of the Most Common Ailments

**Figure 1:**
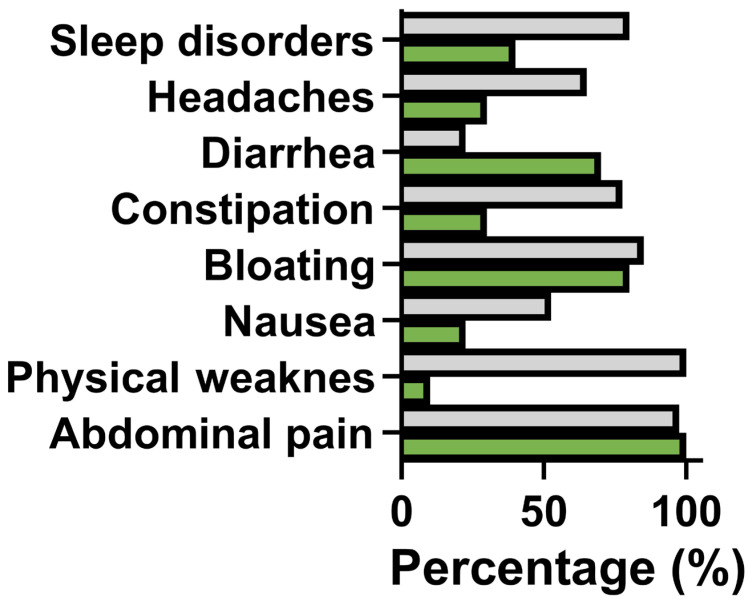
Frequency of symptoms significantly affecting quality of life, as assessed by patients with unclassified irritable bowel syndrome (IBS-U, Group I, green) and patients with chronic fatigue syndrome (IBS-CFS, Group II, grey). All patients reported abdominal pain of a visceral nature. Physical fatigue was significantly more prevalent and severe in Group II (97.5%) compared to Group I (10.0%), where it occurred in milder forms. Constipation was more common in Group II (77.5%), whereas diarrhea was more prevalent in Group I (70.0%). Symptoms such as nausea, bloating, headaches (including chronic migraine), and sleep disorders were also more frequently reported in Group II than in Group I. The score on the Chalder Fatigue Scale in Group II ranged from 16 to 29 points (23.2 ± 8.6). The results of the fatigue severity scale in Group II were from 28 to 54 points (37.1 ± 16.9).

All patients in both groups reported visceral-type abdominal pain. In Group II (IBS-CFS), 12 patients additionally reported chronic abdominal wall pain and nociplastic musculoskeletal pain. No patient reported odynophagia or tender lymph nodes (criteria from the Chalder Scale); however, all Group II patients met the remaining fatigue-related criteria. Physical fatigue was experienced by 97.5% of Group II patients compared to 10% of Group I patients (*p* < 0.001). Fatigue in Group I was mild and did not impact quality of life. Sleep disorders were twice as frequent in Group II compared to Group I (80.0% vs. 40.0%, respectively; *p* < 0.01). Constipation predominated in Group II (77.5%), whereas diarrhea was more common in Group I (70.0%). Headaches diagnosed as chronic migraines were reported by 2 patients in Group I and 4 in Group II. The severity of fatigue was assessed using two validated instruments: the Chalder Fatigue Scale (CFQ-11) and the fatigue severity scale (FSS). In patients from Group II (IBS-CFS), the CFQ-11 scores ranged from 16 to 32 points, with a median value of 27.5 points (interquartile range [IQR]: 21–31), indicating a high burden of fatigue symptoms. Similarly, the FSS scores in Group II ranged from 23 to 53 points, with a median of 37.5 points (IQR: 31–46), confirming the significant impact of fatigue on daily functioning. Physical fatigue was significantly more frequent and severe in Group II (97.5%) compared to Group I (20.0%), where it was mild and did not impair quality of life. These findings highlight the greater severity and functional impact of fatigue in patients with coexisting chronic fatigue syndrome (Figure 1).

### 3.3. Comparison of the Laboratory Data

No statistically significant differences were found in laboratory tests, particularly in those elements that may influence the symptom of fatigue, such as vitamin levels, blood glucose levels, and thyroid-stimulating hormone. Renal and hepatic function parameters are also within normal limits. Similarly, inflammatory markers, including C-reactive protein and calprotectin, were within the routine laboratory ranges for healthy individuals in both groups (Table 1).

### 3.4. Comparison of Breath Hydrogen-Methane Test Results

Basal hydrogen and methane concentrations in breath air were similar in both groups. At 90 min, the increase in hydrogen concentration was higher in Group I; however, in both groups, it exceeded 20 ppm, which is the criterion for SIBO diagnosis. At 90 and 180 min, statistically higher methane concentrations were found in Group II compared to Group I (Table 2).

### 3.5. Gut Microbiome Profile Composition

All patients had an altered microbiome profile compared to the reference group, but the dysbiosis index was similar in both groups. However, the Shannon alpha diversity index value was significantly higher in group II (Table 3).

In both groups, the composition of the gut microbiome was complex. The same bacteria in individual patients showed either an increase or decrease of abundance. In Group I several taxonomic differences were detected including increased relative abundance of *Bifidobacterium* spp., *Bacteroidetes* spp., and *Lactobacillus* spp. in comparison to Group II (Table 4). The significant difference between groups was only related to the increased abundance of Bacteroidetes. For the remaining bacteria the differences were not statistically significant (Table 4).

### 3.6. Comparison of Urinary Phenylalanine and Tryptophan Metabolite Concentrations

**Figure 2:**
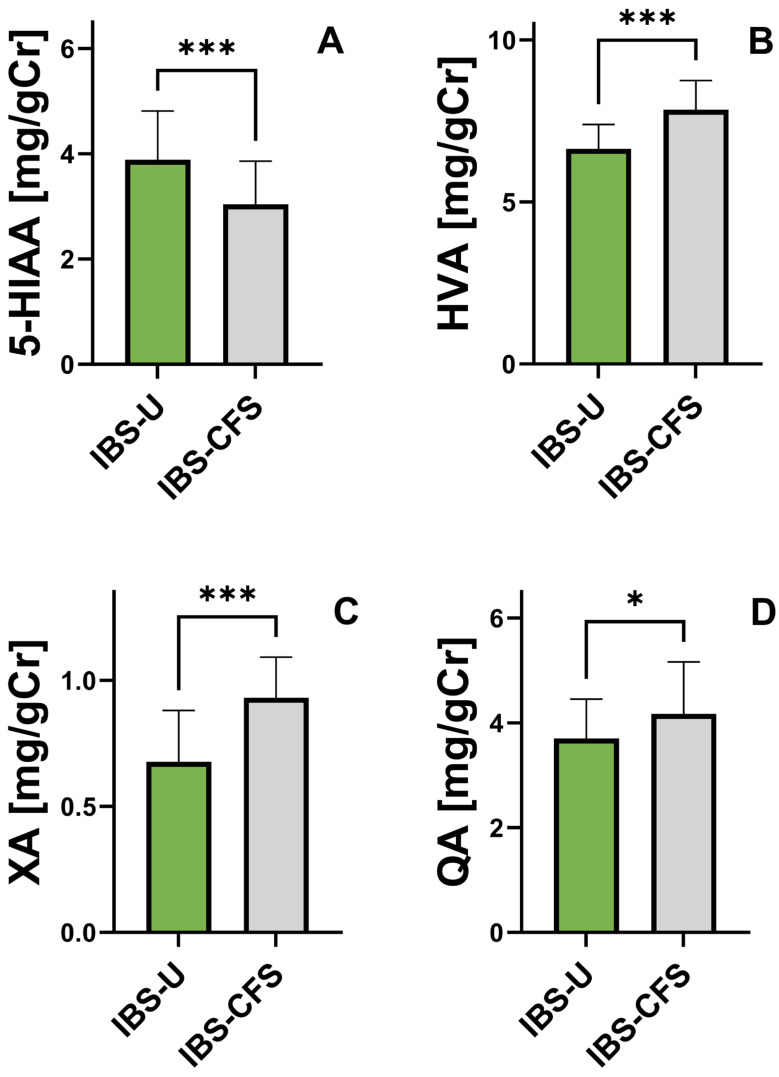
Comparison of urinary concentrations of phenylalanine and tryptophan metabolites in patients with unclassified irritable bowel syndrome (IBS-U) and irritable bowel syndrome coexisting with chronic fatigue syndrome (IBS-CFS). Metabolites analyzed include 5-hydroxyindoleacetic acid (5-HIAA, **A**), homovanillic acid (HVA, **B**), xanthurenic acid (XA, **C**), and quinolinic acid (QA, **D**). Data are presented as mean values ± SD. Statistically significant differences between groups were assessed using the Mann–Whitney U test (* *p* < 0.05, *** *p* < 0.001).

A comparison of urinary concentrations of phenylalanine and tryptophan metabolites revealed significant differences between the IBS-U and IBS-CFS groups (Figure 2). Patients with IBS-CFS exhibited significantly higher median levels of 3-indoxyl sulfate (86.2 vs. 73.6 mg/gCr, *p* < 0.001), homovanillic acid (7.8 vs. 6.7 mg/gCr, *p* < 0.001), quinolinic acid (4.16 vs. 3.69 mg/gCr, *p* = 0.027), and xanthurenic acid (0.935 vs. 0.650 mg/gCr, *p* < 0.001) compared to IBS-U patients. Conversely, lower concentrations of 5-hydroxyindoleacetic acid (3.15 vs. 3.95 mg/gCr, *p* < 0.001) and kynurenine (0.525 vs. 0.570 mg/gCr, *p* = 0.030) were observed in the IBS-CFS group. No significant differences were found for kynurenic acid (KYNA) and hydroxyphenylacetic acid (HPA) between the groups. These results indicate a dysregulation of tryptophan metabolism pathways, particularly along the kynurenine and indole routes, in patients with IBS and coexisting chronic fatigue syndrome. 

### 3.7. Assessment of the Relationship Between Concentration of the Studied Metabolites and Intensity of CFS

**Figure 3:**
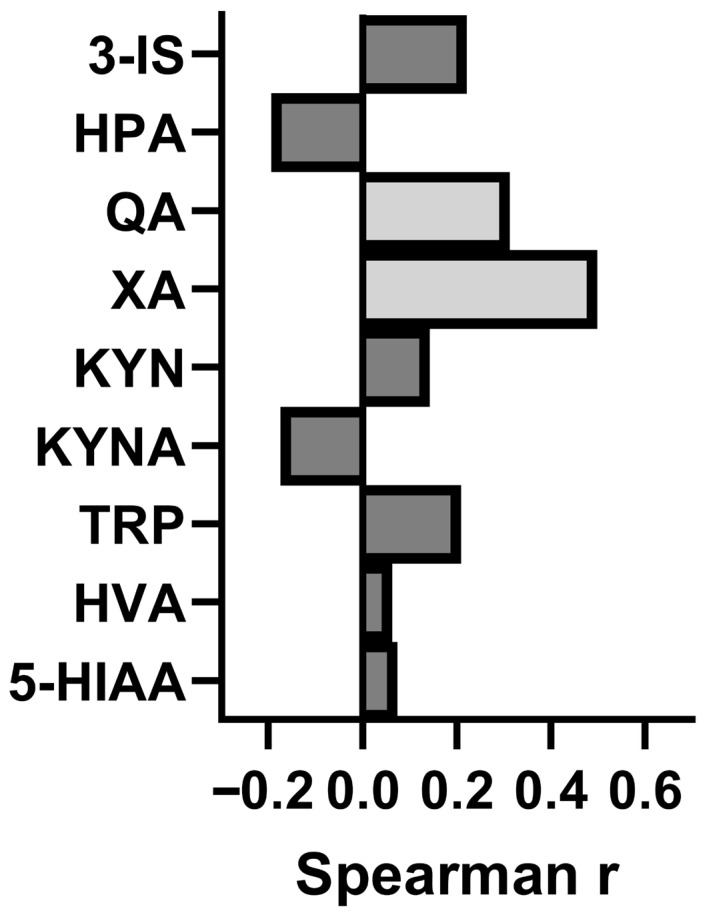
Correlation between urinary concentrations of phenylalanine and tryptophan metabolites and fatigue severity scale (FSS) scores in patients with irritable bowel syndrome coexisting with chronic fatigue syndrome (IBS-CFS). Metabolites analyzed include 3-indoxyl sulfate (3-IS), hydroxyphenylacetic acid (HPA), quinolinic acid (QA), xanthurenic acid (XA), kynurenine (KYN), kynurenic acid (KYNA), tryptophan (TRP), homovanillic acid (HVA), and 5-hydroxyindoleacetic acid (5-HIAA). Correlation coefficients were calculated using Spearman’s rank correlation test. Statistically significant correlations are indicated by light grey.

A correlation analysis was conducted between the urinary concentrations of phenylalanine and tryptophan metabolites and fatigue severity scale (FSS) scores, utilizing Spearman’s rank correlation test (Figure 3). A significant positive correlation was identified between FSS scores and urinary concentrations of xanthurenic acid (Xa) (rho = 0.4995, *p* = 0.001), indicating that higher levels of Xa are associated with greater fatigue severity. Additionally, a weaker but statistically significant positive correlation was observed between quinolinic acid (QA) levels and FSS scores (rho = 0.3129, *p* = 0.049). No significant correlations were found between FSS scores and the concentrations of other metabolites, including 3-indoxyl sulfate (3-IS), 5-hydroxyindoleacetic acid (5-HIAA), homovanillic acid (HVA), kynurenine (KYN), kynurenic acid (KYNA), and hydroxyphenylacetic acid (HPA). These associations do not establish causality and may reflect common underlying pathways or confounding factors.

### 3.8. Post-Hoc Power Analysis

To evaluate the statistical sensitivity of our study, we conducted a post-hoc power analysis based on the observed effect sizes (Cohen’s d) for key urinary metabolites. The effect sizes for the most discriminative variables were as follows: 3-IS: d = 2.19, XA: d = 2.48; HVA: d = 1.29 and QA: d = 0.92. For our sample size (n = 40 per group, α = 0.05), the statistical power for these effects was consistently above 99%, confirming that the study was well-powered to detect large and biologically meaningful group differences. Only KYN exhibited a small effect size (d ≈ 0.39), and its group difference did not survive multiple testing correction. This reinforces the interpretation that our analysis was sufficiently powered for primary outcomes but may be less sensitive to minor metabolic changes. These findings validate the robustness of our main comparisons while transparently delineating the limits of detection for lower-magnitude effects.

Full statistical details, including raw group means, corrected and uncorrected *p*-values, effect sizes, and 95% confidence intervals for all metabolite and breath test comparisons, are provided in Supplementary Tables S1–S3. Individual-level variability in urinary metabolite concentrations is additionally visualized in Supplementary Figure S1 (heatmap).

## 4. Discussion

### 4.1. Microbiome Composition in IBS-CFS: Comparison with Literature

Changes in the gut microbiome have been observed in IBS patients in various studies, but the results obtained are diverse. The most frequently reported findings include an increased abundance of *Enterobacteriaceae*, *Bacteroidetes*, and *Firmicutes* [15,40], along with a decreased abundance of *Bifidobacteria* and *Lactobacilli* [41,42]. Results depend on the type of IBS. In patients with IBS-D, bacteria from the Bacteroidetes, Firmicutes, and Enterobacteriaceae were dominant [43,44], while the numbers of *Bifidobacteria*, *Lactobacilli*, and *Firmicutes prausnitzii* were lower [24,45]. On the other hand, in IBS-C patients, higher levels of *Firmicutes*, *Bacteroidetes*, and *Bifidobacteria* were found [46,47]. The results of some microbiome testing in patients with IBS-M depend on the period of this syndrome [48]. The currently presented study was conducted regardless of the type of abdominal complaints and during a period of severe psychosomatic fatigue. For this reason, the results should be related to the severity of chronic fatigue and similar studies conducted by other researchers. One of these studies indicated a higher level of *Enterococcus* and *Streptococcus* in this syndrome [49,50], while others showed an increasing abundance of Enterobacteriaceae and a decreased number of anti-inflammatory Firmicutes, including *Faecalibacterium prausnitzii* [9,15,51]. In a meta-analysis, it was demonstrated that the abundance of the *Firmicutes phylum*, specifically the genera *Faecalibacterium*, *Roseburia*, and *Clostridium*, is increasing, which is typically associated with CFS [8]. In simple terms, it can be assumed that in IBS patients, there are increased pro-inflammatory and decreased anti-inflammatory bacteria. Our results only partially confirm these opinions because the increases in the abundance of pro-inflammatory bacteria, such as *Clostridium* spp., *Escherichia* spp., and *Ruminococcus varians*, did not differ significantly between the two groups. However, in the group with chronic fatigue, the abundance of anti-inflammatory bacteria, such as *Bacteroidetes* spp., was relatively decreased. The abundance of other anti-inflammatory bacteria, such as *Faecalibacterium prausnitzii* or *Akkermansia muciniphila*, was similar in both groups.

### 4.2. Breath Test Findings and Bacterial Overgrowth Patterns

There were also no significant differences in the microbiome profiles between the groups, but the breath test results showed varying degrees of bacterial overgrowth in the groups. When looking for the cause of extraintestinal symptoms of IBS, differences in bacterial metabolites and levels of neurotransmitters should be taken into account. In particular, differences in the proportions of serotonin and dopamine correspond to the function of the gut–brain axis in the examined patients. CFS includes central (brain and mental), peripheral (muscle), and infection-related fatigue. Central fatigue can be explained by the neurochemical mechanisms of tryptophan. The “tryptophan–serotonin hypothesis” assumes that CF results from increased passage of TRP into the brain and thus from higher levels of serotonin in the brain [52,53,54]. However, in the brain, TRP is transformed to a small extent into serotonin, and serotonin produced in the gut does not cross the blood-brain barrier (BBB). For this reason, many researchers disprove the above hypothesis [55,56]. Subsequently, the “tryptophan-kynurenine enhancement hypothesis” has been proposed to explain the mechanism of central fatigue [57,58]. About 90% of TRP is metabolized into the kynurenine pathway. These metabolites, such as kynurenine, kynurenic acid, xanthurenic acid, and quinolinic acid, have neuroprotective and neurotoxic properties. These effects depend, among other factors, on their concentration, but neurotoxic properties are mainly attributed to quinolinic acid. The above metabolites can cross the BBB and disrupt neurotransmission by different mechanisms [58]. Previously, increased levels of quinolinic acid have been found in irritable bowel syndrome [59], as well as in chronic fatigue [60]. Both quinolinic and xanthurenic acids can disturb glutamate, cholinergic, and dopaminergic neurotransmission in the CNS [61,62]. These neurotoxic activities may also affect the peripheral nerves, including the enteric nervous system. In addition, indoxyl sulfate, a metabolite of the indole pathway, has been shown to increase oxidative stress in various cells, including smooth muscle cells [63]. This metabolite primarily has nephrotoxic and cardiotoxic effects. There is no evidence of a neurotoxic effect of indoxyl. However, high activity of tryptophanase enzymes in the presence of tryptophan can affect the decrease of the serotonin pathway. It has been shown that in dysbiotic patients, the production of indoxyl increases significantly [64].

### 4.3. Tryptophan Metabolism Dysregulation and the Microbiota–Gut–Brain Axis

Our results indicate increased activity of the kynurenine pathway in IBS patients, especially in the presence of coexisting CFS. These patients often suffer from sleep disorders, which may also result from an imbalance between the serotonin and kynurenine pathways in the brain. The significantly lower urinary 5-HIAA levels observed in the IBS-CFS group suggest impaired serotonin turnover. This reduction may result from decreased serotonin synthesis, altered degradation dynamics, or preferential metabolic routing of tryptophan toward the kynurenine pathway. Such a shift is consistent with the “tryptophan metabolic switch” described in inflammatory or stress-related conditions, where indoleamine 2,3-dioxygenase (IDO1) activity is upregulated at the expense of tryptophan hydroxylase (TPH)-mediated serotonin production. Given serotonin’s central role in gastrointestinal motility, mood regulation, and circadian signaling, this dysregulation may contribute to the complex symptomatology observed in IBS-CFS. Furthermore, since serotonin is the direct precursor of melatonin, it is plausible that melatonergic signaling is also impaired in this population—potentially contributing to the high prevalence of sleep disorders. Although melatonin was not measured in the present study, future research should incorporate its assessment to better understand the downstream impact of altered serotonin metabolism in IBS-CFS.

This may reflect associations with changes in microbiota composition; however, a direct mechanistic role of specific bacterial taxa cannot be confirmed by the methods used in this study. This is also supported by the increasing levels of hydrogen and methane in the breath. The obtained results do not exclude the participation of bacteria in the limited intestinal inflammatory process and in the initiation of the kynurenine pathway [65]. In addition to inflammatory factors, many others influence the activity of enzymes that regulate the kynurenine pathway, such as glucocorticoids, thyroid hormones, and female sex hormones [66,67,68]. Decreased production of these hormones may be a cause of increased kynurenine levels in the elderly, who often experience symptoms of fatigue and depression [69,70]. Restoring eubiosis is one of the goals of treating many chronic extraintestinal diseases. For this purpose, various probiotic bacteria with anti-inflammatory properties, such as *Bifidobacterium* and *Lactobacillus*, are used [71,72]. These probiotics in IBS patients reduce abdominal complaints, but the elimination of fatigue or depression is less pronounced [47,71].

### 4.4. Interpretation Caveats: Biomarker Associations vs. Mechanistic Significance

The gut microbiome and gut–brain axis have a profound impact on psychosomatic health through multiple mechanisms. These include hormonal pathways mediated by neurotransmitters, immune pathways regulated by cytokines, neuronal pathways involving the vagus nerve, and metabolic pathways involving indole compounds [73,74]. Different bacteria, to varying extents, can affect the levels of dopamine and serotonin, as well as their metabolites, thereby influencing the clinical picture of the studied syndromes. It is not only the quantitative changes in bacteria that are important, but also the proportion between them.

Our results align with previous observations and suggest that alterations in gut microbiota and tryptophan metabolism may be involved in chronic fatigue symptoms. However, these findings should be interpreted as preliminary associations requiring functional and temporal validation. Overproduction of neuroactive tryptophan metabolites exhibits a positive correlation with fatigue severity; however, these remain statistical associations lacking mechanistic confirmation. While the analyzed correlation suggests a moderate statistical association, its clinical significance remains uncertain, and no established threshold defines what constitutes a clinically meaningful change in urinary kynurenine metabolites regarding fatigue.

These metabolic changes may reflect a consequence of dysbiosis, as phenylalanine and tryptophan intake were similar and closely monitored in both groups. Although quinolinic and xanthurenic acids have known neurotoxic properties, their microbial origin and regulatory pathways could not be determined within the scope of this study. Therefore, the associations between microbiota, tryptophan metabolism, and fatigue symptoms should be considered exploratory.

Any potential diagnostic or therapeutic relevance of these findings requires validation in larger cohorts and interventional settings.

### 4.5. Study Limitations and Methodological Considerations

Several interpretative caveats should be considered when evaluating the observed links between gut microbiota, tryptophan metabolism, and fatigue severity in IBS-CFS patients.

First, although our findings align with previous studies suggesting the involvement of the gut–brain axis in psychosomatic disorders, they must be interpreted as preliminary and non-mechanistic. The correlations between neuroactive tryptophan metabolites—such as quinolinic and xanthurenic acids—and fatigue severity scores indicate statistically significant associations, but do not establish causality or define clinical relevance. No established threshold currently defines what constitutes a clinically meaningful change in urinary kynurenine metabolites concerning fatigue.

Second, the observed overproduction of kynurenine pathway metabolites may be a consequence of dysbiosis rather than its cause. PHA and TRP intake were closely monitored and standardized across both study groups, reducing the likelihood that dietary differences explain these findings. However, these biochemical changes may still result from systemic or local factors unrelated to microbiota activity, such as chronic inflammation, neuroendocrine dysregulation, or psychosocial stress.

Third, it is essential to highlight that the gut microbiome exerts its influence through multiple interlinked pathways, including hormonal (neurotransmitters), immune (cytokines), neuronal (vagus nerve), and metabolic (indole compounds) mechanisms. Different bacterial taxa can variably modulate dopamine, serotonin, and their downstream metabolites, influencing fatigue-related symptomatology. However, our study design and methodological constraints do not allow us to determine which bacterial groups, if any, are functionally involved in modulating these neuroactive metabolites. For example, 3-indoxyl sulfate production depends on both microbial tryptophanase-mediated indole generation and host hepatic sulfation, illustrating the dual microbial–host origin of many studied metabolites. Similarly, quinolinic acid and homovanillic acid may reflect interactions between host immune–neuroendocrine pathways and microbial metabolism, rather than being exclusively attributable to gut bacteria.

Furthermore, while the neurotoxic effects of quinolinic and xanthurenic acids are well established, their precise microbial origin and regulatory pathways could not be identified within the scope of our approach. The GA-map™ test does not support species-level taxonomic resolution or functional inference, and we therefore cannot link specific taxa to the observed metabolite shifts.

Finally, although moderate statistical correlations were observed, their clinical significance remains uncertain. The absence of defined minimal clinically significant differences (MCIDs) for urinary metabolites limits the interpretation of symptom severity or treatment relevance.

Given these limitations, we interpret the metabolite–symptom associations as exploratory observations that provide a rationale for future research. Validation of their diagnostic or therapeutic relevance will require longitudinal studies, interventional trials, and integration of microbiome and metabolome data at functional resolution.

## 5. Conclusions

This study suggests a pattern of associations between gut microbiota diversity, tryptophan metabolism, and fatigue severity in women with IBS and coexisting chronic fatigue syndrome (CFS). Specifically, elevated urinary levels of neuroactive kynurenine pathway metabolites—quinolinic and xanthurenic acids—were positively associated with fatigue intensity, suggesting that dysregulation of this pathway may contribute to the symptom burden in this population.

However, these findings must be interpreted within the constraints of our methodology. The use of the GA-map™ Dysbiosis Test, while clinically validated, limits taxonomic resolution and precludes functional interpretation of microbiome data. Therefore, observed microbiota–metabolite relationships should be regarded as exploratory associations, not mechanistic conclusions. Likewise, the cross-sectional design does not permit inference regarding the temporal direction or causal relevance of these interactions.

While our results align with the proposed importance of the microbiota–gut–brain axis in the pathophysiology of IBS-CFS, the clinical significance of the observed metabolite changes remains unclear. Currently, no thresholds exist to determine whether the level of kynurenine metabolite elevation indicates a meaningful biological or symptomatic effect.

Future research should prioritize longitudinal cohort designs, incorporate species-level microbiome profiling (e.g., 16S rRNA or shotgun metagenomics), and apply untargeted metabolomics or metatranscriptomics to enable functional pathway analysis. Experimental models, including gnotobiotic animal studies and mechanistic intervention trials, will be critical for clarifying causality and determining whether modulation of microbiota or tryptophan metabolism represents a viable therapeutic avenue. Moreover, they should employ stable isotope-labeled precursors, microbial–metabolite correlation analyses, or gnotobiotic models to resolve the relative contributions of host and microbial processes to neuroactive metabolite production.

In addition to generating hypotheses regarding microbiota–metabolite–symptom interactions, this study also demonstrates the feasibility of integrating urinary metabolomic profiling with standardized microbiome assessment in a clinically relevant IBS-CFS cohort. The combined analytical strategy, despite methodological constraints, allowed for the identification of biologically plausible patterns—such as shifts in kynurenine and indole pathways—that warrant further investigation using higher-resolution and functionally informative methods. These include shotgun metagenomics, untargeted metabolomics, and host–microbe interaction models.

In summary, our study identifies preliminary biomarker–symptom associations that may inform future mechanistic and translational research in the context of IBS-CFS.

## Figures and Tables

**Table 1:** Selected laboratory test results in patients with irritable bowel syndrome (IBS-U, Group I) and with chronic fatigue syndrome (IBS-CFS, Group II).

Feature	Group I (n = 40)	IBS-CFS (n = 40)	*p*-Value
Hb (g/dL)	38.1 ± 6.6	38.3 ± 7.1	0.869
Fe (µg/dL)	93.2 ± 0.9	23.0 ± 1.1	0.067
GFR (mL/min)	98.1 ± 14.9	88.6 ± 18.5	0.061
ALT (µ/L)	15.6 ± 3.5	14.9 ± 3.8	0.059
AST (µ/L)	13.9 ± 2.1	14.3 ± 1.8	0.092
CRP (mg/L)	2.3 ± 1.8	3.1 ± 2.3	0.188
FC (µg/g)	26.6 ± 15.7	30.9 ± 12.8	0.509
TSH (µIU/mL)	2.16 ± 0.6	3.21 ± 0.89	0.863
Vit. D3 (ng/mL)	40.8 ± 3.9	38.6 ± 4.7	0.912
Vit. B12 (pg/L)	389.0 ± 102.3	441 ± 121.2	0.822
HbA1c (%)	5.7 ± 0.4	5.2 ± 0.6	0.752

Hb—hemoglobin, Fe—iron, GFR—glomerular filtration rate, ALT—alanine aminotransferase, AST—aspartate aminotransferase, CRP—C-reactive protein, FC—fecal calprotectin, TSH—thyroid-stimulating hormone, Vit. D3—vitamin 25 OH-D, Vit. B12—methylcobalamine, HbA1c—glycated hemoglobin; differences between groups assessed by Student’s *t*-test were statistically insignificant.

**Table 2:** Concentrations of hydrogen and methane in exhaled air in different time intervals of the hydrogen-methane test in IBS-U patients (Group I, n = 40) and in IBS-CFS patients (Group II, n = 40); average ± SD, differences between groups were assessed by the Student’s *t*-test, * *p* < 0.05, ** *p* < 0.01.

Ions (Time, min)	Group I (ppm)	Group II (ppm)	*p*-Value
Hydrogen (0)	10.9 ± 4.72	9.63 ± 5.31	0.064
Hydrogen (90)	47.2 ± 12.4	31.8 ± 6.76 *	0.049
Hydrogen (180)	79.4 ± 18.2	69.1 ± 16.3	0.052
Methane (0)	3.73 ± 1.62	5.12 ± 2.63	0.058
Methane (90)	8.56 ± 1.19	13.2 ± 3.25 *	0.036
Methane (180)	11.8 ± 2.03	16.4 ± 2.35 **	0.009

**Table 3:** Value of the dysbiosis index (DI) and the Shannon alpha and diversity index (SDI) in IBS-U and IBS-CFS patients; differences between groups were assessed by the Student’s *t*-test or the Mann–Whitney U test, ** *p* < 0.01.

Dysbiosis Indicators	Group I	Group II	*p*-Value
DI (points)	3.69 ± 1.04	3.72 ± 1.28	0.746
SDI (points)	2.07 ± 0.59	2.23 ± 0.51 **	0.003

**Table 4:** Prevalence of increased and decreased bacterial abundance in patients with unclassified irritable bowel syndrome (IBS-U, Group I) and with IBS coexisting with chronic fatigue syndrome (IBS-CFS, Group II). Data are presented as the percentage (%) of patients within each group. Differences in bacterial abundance were assessed using the two-proportion Z-test, * *p* < 0.05.

Bacteria Species	Increase (%), Group I/II	*p*-Value	Decrease (%) Group I/II	*p*-Value
*Bifidobacterium* spp.	42.5/30.0	0.352	22.5/30.0	0.611
*Bacteroidetes* spp.	40.0/15.0	0.024 *	15.0/25.0	0.402
*Lactobacillus* spp.	25.0/10.0	0.141	17.5/35.0	0.127
*Streptococcus* spp.	10.0/10.0	0.709	10.0/15.0	0.735
*Faecalibacterium prausnitzii*	10.0/25.0	0.141	10.0/25.0	0.141
*Akkermansia muciniphila*	5.0/15.0	0.264	5.0/15.0	0.264
*Clostridium* spp.	15.0/10.0	0.735	10.0/22.5	0.365
*Escherichia* spp.	10.0/15.0	0.735	15.0/15.0	0.747
*Prevotella* spp.	20.0/22.5	0.990	10.0/15.0	0.735
*Ruminococcus* v.	10.0/15.0	0.735	27.5/25.0	0.999

## Data Availability

The data from this study are available to be shared upon reasonable request.

## Supplementary Materials

The following supporting information can be downloaded at: https://www.mdpi.com/article/10.3390/nu17132232/s1, Table S1. Breath Test Gas Concentrations (HMBT). Table S2. Key Metabolites with Significant or Borderline Differences. Table S3. Metabolites Without Statistically Significant Differences. Figure S1. Heatmap visualization of individual urinary metabolite concentrations across study participants.

## References

[1] Brurberg K.G., Fønhus M.S., Larun L., Flottorp S., Malterud K. (2014). Case definitions for chronic fatigue syndrome/myalgic encephalomyelitis (CFS/ME): A systematic review. BMJ Open.

[2] Morris G., Maes M., Berk M., Puri B.K. (2019). Myalgic encephalomyelitis or chronic fatigue syndrome: How could the illness develop. Metabol. Brain Dis..

[3] Holmes G.P. (1988). Chronic Fatigue Syndrome: A Working Case Definition. Ann. Intern. Med..

[4] Fukuda K., Straus S.E., Hickie I., Sharpe M.C., Dobbins J.G., Komaroff A. (1994). The Chronic Fatigue Syndrome: A Comprehensive Approach to Its Definition and Study. Ann. Intern. Med..

[5] Carruthers B.M., Jain A.K., De Meirleir K.L., Peterson D.L., Klimas N.G., Lerner A.M., Bested A.C., Flor-Henry P., Joshi P., Powles A.C.P. (2003). Myalgic Encephalomyelitis/Chronic Fatigue Syndrome: Clinical Working Case Definition, Diagnostic and Treatment Protocols. J. Chronic Fatigue Syndr..

[6] Committee on the Diagnostic Criteria for Myalgic Encephalomyelitis/Chronic Fatigue Syndrome, Board on the Health of Select Populations and Institute of Medicine (2015). Beyond Myalgic Encephalomyelitis/Chronic Fatigue Syndrome: Redefining an Illness.

[7] Tate W., Walker M., Sweetan E., Heliweli A., Peppercorn K., Edgar C., Blair A., Chatterjee A. (2022). Molecular Mechanism of Neuroinflommaton in ME/CFS snd Long COVID to Sustain Disease and Promote Relapses. Front. Neurol..

[8] Nagy-Szakal D., Williams B.L., Mishra N., Che X., Lee B., Bateman L., Klimas N.G., Komaroff A.L., Levine S., Montoya J.G. (2017). Fecal Metagenomic Profiles in Subgroups of Patients With Myalgic Encephalomyelitis/Chronic Fatigue Syndrome. Microbiome.

[9] Fremont M., Coomans D., Massart S., De Meirleir K. (2013). High-Throughput 16S rRNA Gene Sequencing Reveals Alterations of Intestinal Microbiota in Myalgic Encephalomyelitis/Chronic Fatigue Syndrome Patients. Anaerobe.

[10] Armstrong C.W., McGregor N., Lewis D.P., Butt H.L., Gooley P.R. (2017). The Association of Fecal Microbiota and Fecal, Blood Serum and Urine Metabolites in Myalgic Encephalomyelitis/Chronic Fatigue Syndrome. Metabolomics.

[11] Navaneetharaja N., Griffiths V., Wileman T., Carding S. (2016). A Role for the Intestinal Microbiota and Virome in Myalgic Encephalomyelitis/Chronic Fatigue Syndrome (ME/CFS)?. J. Clin. Med..

[12] Maes M., Mihaylova I., Leunis J.-C. (2007). Increased Serum IgA and IgM Against LPS of Enterobacteria in Chronic Fatigue Syndrome (CFS): Indication for the Involvement of Gram-Negative Enterobacteria in the Etiology of CFS and for the Presence of an Increased Gut–Intestinal Permeability. J. Affect. Disord..

[13] Lupo G.F.D., Rocchetti G., Lucini L., Lorusso L., Manara E., Bertelli M., Puglisi E., Capelli E. (2021). Potential Role of Microbiome in Chronic Fatigue Syndrome/Myalgic Encephalomyelits (CFS/ME). Sci. Rep..

[14] Hanson M.R., Giloteaux L. (2017). The Gut Microbiome in Myalgic Encephalomyelitis. Biochemist.

[15] Maes M., Twisk F.N., Kubera M., Ringel K., Leunis J.-C., Geffard M. (2012). Increased IgA Responses to the LPS of Commensal Bacteria Is Associated With Inflammation and Activation of Cell-Mediated Immunity in Chronic Fatigue Syndrome. J. Affect. Disord..

[16] Barker E., Fujimura S.F., Fadem M.B., Landay A.L., Levy J.A. (1994). Immunologic Abnormalities Associated With Chronic Fatigue Syndrome. Clin. Infect. Dis..

[17] Lakhan S.E., Kirchgessner A. (2010). Gut Inflammation in Chronic Fatigue Syndrome. Nutr. Metab..

[18] Berstad A., Hauso O., Berstad K., Berstad J.E.R. (2020). From IBS to ME—The Dysbiotic March Hypothesis. Med. Hypotheses.

[19] El-Salhy M. (2023). Intestinal bacteria associated with irritable bowel syndrome and chronić fatigue syndrome. Neurogastroenterol. Motil..

[20] Galland L. (2014). The Gut Microbiome and the Brain. J. Med. Food.

[21] Raij T., Raij K. (2024). Association between fatigue, peripheral serotonin, and L-carnitine in hypothyroidism and in chronic fatigue syndrome. Front. Endocrinol..

[22] Gao J., Xu K., Liu H., Liu G., Bai M., Peng C., Li T., Yin Y. (2018). Impact of the Gut Microbiota on Intestinal Immunity Mediated by Tryptophan Metabolism. Front. Cell Infect. Microbiol..

[23] Roager H.M., Licht T.R. (2018). Microbial Tryptophan Catabolites in Health and Disease. Nat. Commun..

[24] Kashi A.A., Davis R.W., Phair R.D. (2019). The IDO Metabolic Trap Hypothesis for the Etiology of ME/CFS. Diagnostics.

[25] Laurans L., Venteclef N., Haddad Y., Chajadine M., Alzaid F., Metghalchi S., Sovran B., Denis R.G.P., Dairou J., Cardellini M. (2018). Genetic Deficiency of Indoleamine 2,3-Dioxygenase Promotes Gut Microbiota-Mediated Metabolic Health. Nat. Med..

[26] Liu W.-l., Lin Y.-H., Xiao H., Xing S., Chen H., Chi P.-D., Zhang G., Longnecker R.M. (2014). Epstein-Barr Virus Infection Induces Indoleamine 2,3-Dioxygenase Expression in Human Monocyte-Derived Macrophages Through P38/Mitogen-Activated Protein Kinase and NF- B Pathways: Impairment in T Cell Functions. J. Virol..

[27] Magro F., Vieira-Coelho M.A., Fraga S., Serrao M.M., Veloso F.T., Robeiro T., Soares da Silva P. (2002). Impaired synthesis or cellular storage of norepinephrine, dopamine, and 5-hydroxytryptamine in human inflammatory bowel diseases. Dig. Dis. Sci..

[28] Strandwitz P. (2018). Neurotransmitterr modulation by the gut microbiota. Brain Res..

[29] Yano J.M., Yu K., Donaldson G.P., Shastri G.G., Ann P., Ma L., Nagler C.R., Ismagilov R.F., Mazmanian S.K., Hsiao E.Y. (2015). Indigenous Bactria of Gut Microbiota Regulate Serotonin Biosynthesis. Cell.

[30] Zhang Y.J., Gran R.Y., Zhou T., Xu D.P., Li H.B. (2015). Impact of gut bacteria on human health and diseases. Int. J. Mol. Sci..

[31] Wang J.-K., Yao S.-K. (2021). Roles of Gut Microbiota and Metabolites in Pathogenesis of Functional Constipation. Evid.-Based Complement. Altern. Med..

[32] Napolitano M., Fasulo E., Ungaro F., Massimino L., Sinagra E., Danese S., Mandarino F.V. (2023). Gut Dysbiosis in Irritable Bowel Syndrome: A Narrative Review on Correlation with Disease Subtypes and Novel Therapeutic Implications. Microorganisms.

[33] Chen M., Ruan G., Chen L., Ying S., Li G., Xu F., Xiao Z., Tian Y., Lv L., Ping Y. (2021). Naurotransmitter and Intestinal Interactions: Focus on the Microbiota-Gut-Brain Axis in Irritable Bowel Syndrome. Front. Endocrinol..

[34] Lacy B.E., Patel N.K. (2017). Rome Criteria and a Diagnostic Approach to Irritable Bowel Syndrome. J. Clin. Med..

[35] Palsson O.S., Baggish J.S., Turner M.J., Whithead W.E. (2012). Patients Show Frequent Fluctuations between Loose/Watery andHard/Lumpy Stools: Implications for treatment. Am. J. Gastroenterol..

[36] Chalder T., Berelowitz G., Pawlikowska T., Watts L., Wessely S., Wright D., Wallace E.P. (1993). Development of fatigue scale. J. Psychosom. Res..

[37] Krupp L.B., LaRocca N.G., Muir-Nash J., Steiberg A.D. (1989). The fatigue severity scale: Application to patients with multiple sclerosis and systemic lupus erthematosus. Arch. Neurol..

[38] Pimentel M., Saad R., Long M.D., Rao S.S. (2020). ACG Clinical Guideline: Small Intestinal Bacterial Overgrowth. Am. J. Gastroenterol..

[39] Casén C., Vebo H.C., Sekelja M., Hegge F.T., Karlson M.K., Ciemniejewska E., Dzankovic S., Frøyland C., Nestestog R., Engstrand L. (2015). Deviations in human gut microbiota: A novel diagnostic test for determining dysbiosis in patients with IBS or IBD. Aliment. Pharmacol. Ther..

[40] Jackson M.L., Butt H., Ball M., Lewis D.P., Bruck D. (2015). Sleep Quality and the Treatment of Intestinal Microbiota Imbalance in Chronic Fatigue Syndrome: A Pilot Study. Sleep Sci..

[41] Shukla S.K., Cook D., Meyer J., Vernon S.D., Le T., Clevidence D., Robertson C.E., Schrodi S.J., Yale S., Frank D.N. (2015). Changes in Gut and Plasma Microbiome Following Exercise Challenge in Myalgic Encephalomyelitis/Chronic Fatigue Syndrome (ME/CFS). PLoS ONE.

[42] Bested A.C., Logan A.C., Selhub E.M. (2013). Intestinal Microbiota, Probiotics and Mental Health: From Metchnikoff to Modern Advances: Part II—Contemporary Contextual Research. Gut Pathog..

[43] Wallis A., Ball M., McKechnie S., Butt H., Lewis D.P., Bruck D. (2017). Examining Clinical Similarities Between Myalgic Encephalomyelitis/Chronic Fatigue Syndrome and D-Lactic Acidosis: A Systematic Review. J Transl. Med..

[44] Russell A., Hepgul N., Nikkheslat N., Borsini A., Zajkowska Z., Moll N., Forton D., Agarwal K., Chalder T., Mondelli V. (2019). Persistent Fatigue Induced by Interferon-Alpha: A Novel, InflammationBased, Proxy Model of Chronic Fatigue Syndrome. Psychoneuroendocrinology.

[45] Maier L., Pruteanu M., Kuhn M., Zeller G., Telzerow A., Anderson E.E., Brochado A.R., Fernandez K.C., Dose H., Mori H. (2018). Extensive Impact of Non-Antibiotic Drugs on Human Gut Bacteria. Nature.

[46] Kenyon J.N., Coe S., Izadi H. (2019). A Retrospective Outcome Study of 42 Patients With Chronic Fatigue Syndrome, 30 of Whom had Irritable Bowel Syndrome. Half Were Treated With Oral Approaches, and Half Were Treated with Faecal Microbiome Transplantation. Hum. Microbiome J..

[47] Corbitt M., Campagnolo N., Staines D., Marshall-Gradisnik S. (2018). A Systematic Review of Probiotic Interventions for Gastrointestinal Symptoms and Irritable Bowel Syndrome in Chronic Fatigue Syndrome/Myalgic Encephalomyelitis (CFS/ME). Probiotics Antimicrob. Proteins.

[48] Chojnacki J., Konrad P., Mędrek-Socha M., Kaczka A., Błońska A., Zajdel R., Chojnacki C., Gąsiorowska A. (2023). The Variability of Tryptophan Metabolism in Patients With Mixed Type of Irritable Bowel Syndrome. Int. J. Mol. Sci..

[49] Sheedy J.R., Wettenhall R.E.H., Scanlon D., Gooley P.R., Lewis D.P., McGregor N., I Stapleton D., Butt H.L., De Meirleir K.L. (2009). Increased D-Lactic Acid Intestinal Bacteria in Patients With Chronic Fatigue Syndrome. In Vivo.

[50] Wallis A., Ball M., Butt H., Lewis D.P., McKechnie S., Paull P., Jaa-Kwee A., Bruck D. (2018). Open-Label Pilot for Treatment Targeting Gut Dysbiosis in Myalgic Encephalomyelitis/ Chronic Fatigue Syndrome: Neuropsychological Symptoms and Sex Comparisons. J. Transl. Med..

[51] Giloteaux L., Goodrich J.K., Walters W.A., Levine S.M., Ley R.E., Hanson M.R. (2016). Reduced Diversity and Altered Composition of the Gut Microbiome in Individuals With Myalgic Encephalomyelitis/Chronic Fatigue Syndrome. Microbiome.

[52] Newsholme E.A., Blomstrand E. (2006). Branched-chain amino acids and central fatigue. J. Nutr..

[53] Fernstrom J.D., Fernstrom M.H. (2006). Exercise, serum free tryptophan, and central fatigue. J Nutr..

[54] Yamashita M., Yamamoto T. (2014). Tryptophan and kynurenic acid may produce an amplified effect in central fatigue induced by chronic sleep disorder. Int. J. Tryptophan Res..

[55] Yamamoto T., Azechi H., Board M. (2012). Essential role of excessive tryptophan and its neurometabolites in fatigue. Can. J. Neurol. Sci..

[56] Yamashita M., Yamamoto T. (2017). Tryptophan circuit in fatigue: From blood to brainand cognition. Brain Res..

[57] Åkesson K., Pettersson S., Ståhl S., Surowiec I.M. (2018). Kynurenine pathway is altered in patients with SLE and associated with severe fatigue. Lupus Sci. Med..

[58] Németh H., Toldi J., Vécsei L. (2005). Role of kynurenines in the central and peripheral nervous systems. Curr. Neurovasc. Res..

[59] Chojnacki C., Błońska A., Konrad P., Chojnacki M., Podogrocki M., Popławski M. (2023). Changes in Tryptophan Metabolism on serotonin and Kynurenine Pathways in Patients with Irritable Bowel Syndrome. Int. J. Mol. Sci..

[60] Lugo-Huitrón R., Muñiz P.U., Pineda B., Pedraza-Chaverrí J., Ríos C., Pérez-de la Cruz V. (2013). Quinolinic acid: An endogenous neurotoxin with multiple target. Oxid. Med. Cell Longev..

[61] Carpenedo R., Pittaluga A., Cozzi A., Attuci S., Galli A., Moroni F. (2001). Presynaptic kynurenate-sensitive receptors inhibit glutamate release. Eur. J. Neurosci..

[62] Wu H.Q., Pereira E.F., Bruno J.P., Pellicciari R., Albuquerque E.X., Schwarcz R. (2010). The astrocyte-derived alpha7 nicotinic receptor antagonist kynurenic acid controls extracellular glutamate levels in the prefrontal cortex. J. Mol. Neurosci..

[63] Guo J., Lu L., Hua Y., Huang K., Wang I., Huang L. (2017). Vasculopathy in the setting of cardiorenal syndrome: Roles of protein-bound uremic toxins. Am. J. Physiol. Heart Circ. Physiol..

[64] Lord R.S., Bralley J.A. (2008). Clinical applications of urinary organic acids. Part 2. Dysbiosis markers. Altern. Med. Rev..

[65] Chong P.P., Chin V.K., Looi C.Y., Wong W.F., Madhavan P., Yong V.C. (2019). The Microbiome and Irritable Bowel Syndrome—A Review on the Pathology, Current Research and Future Therapy. Front. Microbiol..

[66] Jovanovic F., Jovanovic V., Knezevic N.N. (2023). Glucocoricoid Hormones as Modulators of the Kynurenine Pathway in Chronic Pain Conditions. Cells.

[67] Tomczyk T., Urbańska E.M. (2020). Experimental hypothyroidism raises brain kynurenic acid—Novel aspect of thyroid dysfunction. Eur. J. Pharmacol..

[68] Meier T.B., Drevets W.C., Teague T.K., Wurfl B.E., Muller S.C., Bodurka J., Dantzer R., Savitz J. (2018). Kynurenic acid is reduced in females and contraceptive users: Implications for depression. Brain Behav. Immun..

[69] Bakker L., Choe K., Eussen S.J.P.M., Ramarkes I.H.G.B., van den Hove D.L.A., Kenis G., Rutten B.P.F., Verhey F.R.J., Köhler S. (2024). Relation of the kynurenine pathway with normal age: A systemic review. Mech. Ageing Dev..

[70] Dehhaghi M., Panahi H.K.S., Kavyani B., Heng B., Tan V., Braidy N., Guillemin G.J. (2022). The Role of Kynurenine Pathway and NAD+ Metabolsm in Myalgic Encephalomyelitis/ Chronic Fatigue Syndrome. Aging Dis..

[71] Sullivan Å., Nord C.E., Evengård B. (2009). Effect of Supplement With Lactic-Acid Producing Bacteria on Fatigue and Physical Activity in Patients With Chronic Fatigue Syndrome. Nutr. J..

[72] Rondanelli M., Faliva M.A., Perna S., Giacosa A., Peroni G., Castellazzi A.M. (2017). Using Probiotics in Clinical Practice: Where Are We Now? A Review of Existing Meta-Analyses. Gut Microbes.

[73] Mayer E.A. (2011). Gut Feelings: The Emerging Biology of Gut–Brain Communication. Nat. Rev. Neurosci..

[74] Lin X., Yu Z., Liu Y., Li C., Hu H., Hu J.-C., Liu M., Yang Q., Gu P., Li J. (2025). Gut-X axis. Imeta.

