# Supplementary material for: Assessment of the Gut Microbiome in Patients with Coexisting Irritable Bowel Syndrome and Chronic Fatigue Syndrome
Source: Nutrients. 2025 Jul 5;17(13):2232. doi: 10.3390/nu17132232 (PMC12251473; doi:10.3390/nu17132232)
Supplement: Supplementary file 1 [file nutrients-17-02232-s001.zip › nutrients-3685257-supplementary.pdf]

Supplementary file for manuscript

Assessment of the gut microbiome in patients with coexisting irritable bowel syndrome and chronic fatigue syndrome

Marcin Chojnacki, Aleksandra Błońska, Aleksandra Kaczka, Jan Chojnacki, Ewa Walecka-Kapica, Natalia Romanowska, Karolina Przybylowska-Sygut, Tomasz Popławski

Supplementary Tables – Metabolite Group Comparisons

Supplementary Table S1. Breath Test Gas Concentrations (HMBT)

Comparison of exhaled hydrogen and methane concentrations at baseline and post-lactulose ingestion between IBS-U and IBS-CFS groups. Uncorrected and Bonferroni-adjusted p-values (α = 0.0083 for 6 tests) are provided. Values with p < 0.0083 would be considered statistically significant. All findings should be interpreted as exploratory trends due to modest effect sizes and borderline significance.

Timepoint & Gas	Mean (IBS-U)	Mean (IBS-CFS)	p (uncorr.)	p (Bonf.)	Interpretation
Hydrogen (0 min)	10.9	9.63	0.064	n.s.	Not significant
Hydrogen (90 min)	47.2	31.8	0.049	n.s.	Trend only
Hydrogen (180 min)	79.4	69.1	0.052	n.s.	Trend only
Methane (0 min)	3.73	5.12	0.058	n.s.	Not significant
Methane (90 min)	8.56	13.2	0.036	n.s.	Trend only
Methane (180 min)	11.8	16.4	0.009	borderline	Trend only

Supplementary Table S2. Key Metabolites with Significant or Borderline Differences

Comparison of urinary metabolite concentrations between IBS-U and IBS-CFS groups. Shown are group means, uncorrected and Bonferroni-corrected p-values (adjusted α = 0.0056 for 9 tested metabolites), effect sizes (Cohen's d), and estimated 95% confidence intervals for mean differences. Metabolites with p < 0.0056 after correction are considered statistically significant. Other findings are reported as exploratory trends.

Metabolite	Mean (IBS-U)	Mean (IBS-CFS)	p (uncorr.)	p (Bonf.)	Cohen's d	95% CI (mean diff.)	Interpretation
3-indoxyl sulfate	73.6	86.2	<0.001	<0.001	2.19	+12.6 to +16.4	Significant
Xanthurenic acid	0.65	0.935	<0.001	<0.001	2.48	+0.23 to +0.34	Significant
Homovanillic acid	6.7	7.8	<0.001	<0.001	1.29	+0.86 to +1.38	Significant
Quinolinic acid	3.69	4.16	0.027	n.s.	0.92	+0.12 to +0.85	Trend only

Kynurenine	0.57	0.525	0.030	n.s.	0.39	-0.09 to -0.01	Trend only

Supplementary Table S3. Metabolites Without Statistically Significant Differences

Comparison of urinary concentrations of tryptophan and phenylalanine pathway metabolites that showed no statistically significant differences between IBS-U and IBS-CFS groups. All p-values remained above the Bonferroni-adjusted threshold (α = 0.0056). Effect sizes were small to negligible.

Metabolite	Mean (IBS-U)	Mean (IBS-CFS)	p (uncorr.)	p (Bonf.)	Cohen's d	95% CI (mean diff.)	Interpretation
5-HIAA	3.95	3.15	0.12	n.s.	0.35	-0.25 to +0.85	Not significant
Kynurenic acid	0.12	0.11	0.21	n.s.	0.15	-0.01 to +0.03	Not significant
Tryptophan	15.1	15.3	0.77	n.s.	0.05	-1.1 to +1.5	Not significant
Hydroxyphenylacetic acid	1.23	1.19	0.44	n.s.	0.1	-0.08 to +0.15	Not significant

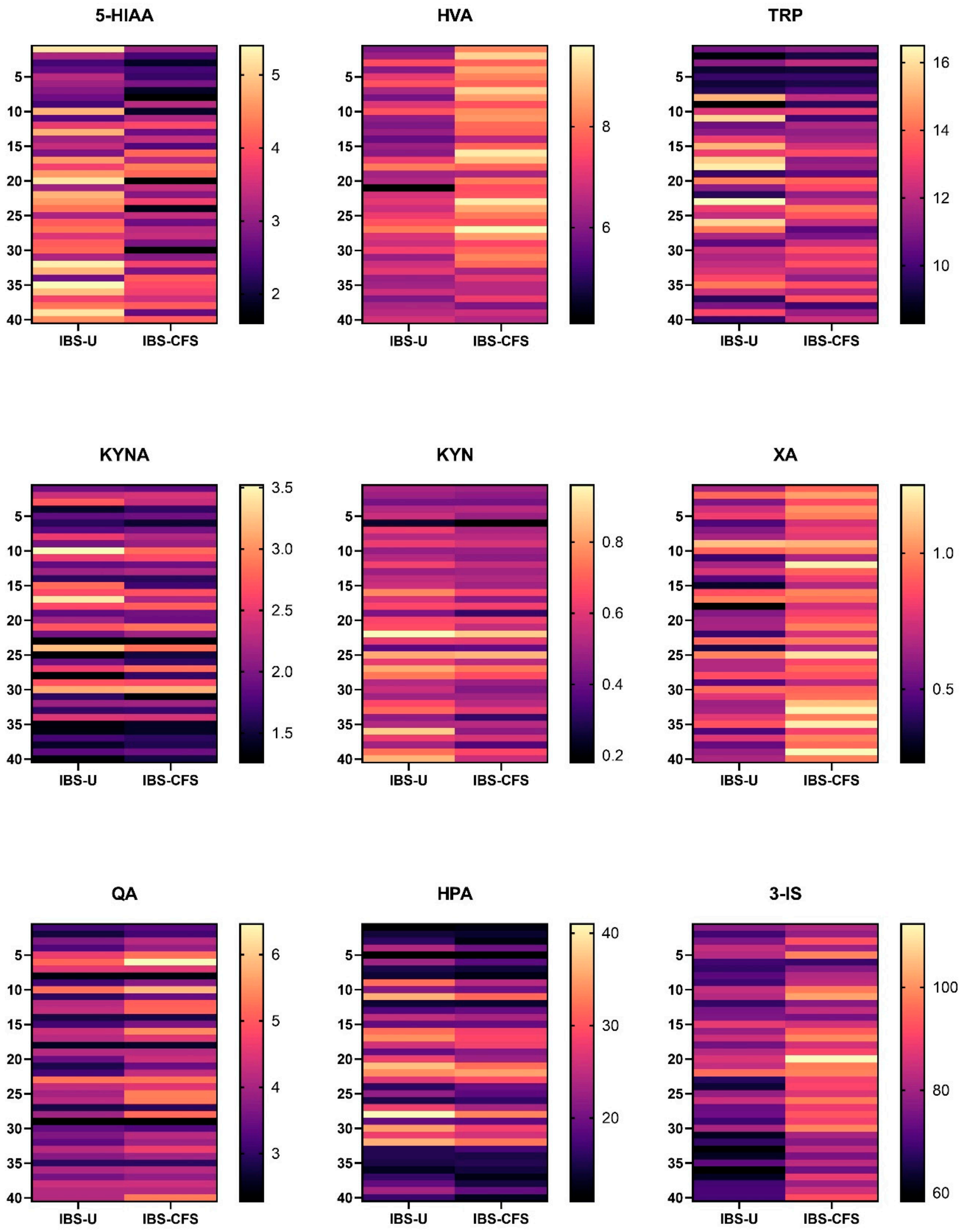

Supplementary Figure S1. Heatmap visualization of individual urinary metabolite concentrations across study participants. Each row represents one participant, grouped by IBS-U (n = 40) or IBS-CFS (n = 40). Columns correspond to nine urinary metabolites from tryptophan and phenylalanine pathways. Color intensity reflects values scaled per metabolite (min–max normalization within each column), with warmer tones (Magma gradient) indicating higher relative concentrations. This visualization highlights interindividual heterogeneity and group-level clustering patterns.
